# Activation of Mitochondrial Unfolded Protein Response in SHSY5Y Expressing APP Cells and APP/PS1 Mice

**DOI:** 10.3389/fncel.2019.00568

**Published:** 2020-01-08

**Authors:** Yang Shen, Mao Ding, Zhaohong Xie, Xiangtian Liu, Hui Yang, Suqin Jin, Shunliang Xu, Zhengyu Zhu, Yun Wang, Dewei Wang, Linlin Xu, Xiaoyan Zhou, Ping Wang, Jianzhong Bi

**Affiliations:** ^1^Medicine School, Shandong University, Jinan, China; ^2^Department of Neurology Medicine, Second Hospital of Shandong University, Jinan, China

**Keywords:** Alzheimer disease, UPRmt, mevalonate pathway, sphingolipid biosynthesis pathway, simvastatin

## Abstract

Alzheimer disease (AD) is the most common form of dementia. Amyloid β-peptide (Aβ) deposition is a major neuropathologic feature of AD. When unfolded or misfolded proteins accumulate in mitochondria, the unfolded protein responses (UPRmt) is initiated. Numerous lines of evidence show that AD pathogenesis involves mitochondrial dysfunction. However little is known about whether the UPRmt is engaged in the process of AD development. In this study, we investigated the UPRmt in mouse and cell models of AD. We found that UPRmt was activated in the brain of 3 and 9 months old APP/PS1 mice, and in the SHSY5Y cells after exposure to Aβ_25–35_, Aβ_25–35_ triggered UPRmt in SHSY5Y cells could be attenuated upon administration of simvastatin or siRNA for HMGCS-1 to inhibit the mevalonate pathway, and or upon knocking down Serine palmitoyltransferase long chain subunit 1 (SPTLC-1) to lower sphingolipid biosynthesis. We observed that inhibition of UPRmt aggravated cytotoxic effects of Aβ_25–35_ in SHSY5Y cells. Our research suggests that the UPRmt activation and two pathways necessary for this response, and further provides evidence for the cytoprotective effect of UPRmt during the AD process.

## Introduction

Alzheimer disease (AD) is the most common form of dementia in the elderly population and characterized by progressive deterioration of cognitive and functional abilities (Pleckaityte, 2010). Extracellular amyloid plaques, consisting of polymers of amyloid-β peptides (Aβ), and intracellular neurofibrillary tangles, formed mainly by hyperphosphorylated protein tau, are the main neuropathology of AD (Swerdlow, 2007). Diverse lines of evidence support that oligomeric species of Aβ are the causative agent of AD. Aβ is a general term to define 38–43 amino acid peptides generated from the sequential cleavage of the Amyloid Precursor Protein (APP) by β- and γ-secretase (Da Costa Dias et al., 2011). While Aβ_1–40_ and Aβ_1–42_ are the main forms of Aβ peptides deposited in extracellular amyloid plaques, Aβ_1–42_ is more prone to aggregation than Aβ_1–40_ (Selkoe, 2001). The APPsw/PS1dE9 double transgenic mouse line is widely used for investigating the pathogenic mechanisms of AD for its property of effective production of Aβ_1–42_ (Borchelt et al., 1996). Aβ_25–35_, a synthetic peptide of 11 amino acids, retains physical and biological properties of full-length Aβ and is often employed for generating acute AD models (Kang et al., 1987; Harkany et al., 2000).

Mitochondria are crucial cellular organelles essential for numerous cellular functions including energy homeostasis, metabolism, and apoptosis (Wang and Youle, 2009; Blackstone, 2015). Disruption of mitochondrial protein folding homeostasis results in the accumulation of unfolded or misfolded proteins and induce a mitochondria-to-nuclear signal transduction pathway termed the unfolded protein responses (UPRmt), through which upregulated expression of mitochondrial molecular chaperones and proteases is induced to re-establish protein homeostasis (Zhao et al., 2002; Tatsuta and Langer, 2008; Papa and Germain, 2011; Pellegrino et al., 2013). The accumulation of misfolded proteins has been described as a pathological hallmark of numerous neurodegenerative diseases, including AD, Parkinson’s disease, Huntington’s disease and amyotrophic lateral sclerosis (Skovronsky et al., 2006). Although Aβ accumulation in mitochondria has long been known to exist in brain of AD patients as well as TgAPP mice (Lustbader et al., 2004; Caspersen et al., 2005; Manczak et al., 2006), little is known about whether the UPRmt is involved in the pathogenesis of AD. A couple of lines of evidence suggest a role for the UPRmt in the development of AD pathology. Levels of HtrA2/Omi (high temperature requirement protein A2/Omi), a protein operating in the process of UPRmt, are reduced in AD frontal cortex, yet its enzymatic activity is significantly increased in the same samples (Westerlund et al., 2011). In addition, there exists increased transcription and translation of UPRmt associated genes in the brain of familial and sporadic AD patients, e.g., upregulation of *hspd1* (HSP60) and *clpp* (CLPP mitochondrial protease) genes, but not *lon1p1* (the LONP1 mitochondrial protease; Beck et al., 2016).

The mevalonate pathway produces isoprenoids, which are vital for diverse cellular functions (Goldstein and Brown, 1990), and has been demonstrated to be required for the activation of the UPRmt in *C. elegans*. 3-hydroxy-3-methyl-glutaryl-CoA (HMG-CoA) reductase is the rate-limiting enzyme in the mevalonate pathway; statins inhibit this enzyme to lower plasma cholesterol levels. Upon treatment with statins, *C. elegans* fail to sense mitochondrial damage and to activate the UPRmt (Liu et al., 2014; Ranji et al., 2014; Oks et al., 2018). Inactivation of the *hmgs-1* gene, which encodes HMG-CoA synthase, renders *C. elegans* to lose the capability to respond to mitochondrial dysfunction and to inhibit antimycin-induced UPRmt induction (Liu et al., 2014). Human hydroxymethylglutaryl-CoA synthase 1 (HMGCS-1) is the ortholog of *C. elegans* HMGS-1 protein and mediates the first committed step of the mevalonate pathway (Sapir et al., 2014). We hypothesize that the mevalonate pathway participates in the activation of UPRmt in the process of AD development.

Sphingolipids are a class of lipids that are highly enriched in the central nervous system and play important functions in membrane structure, signal transduction, and a variety of biological processes (Spiegel and Merrill, 1996; Mielke and Haughey, 2012). Alterations in the sphingolipids metabolism are thought to be concerned with AD development. The rate-limiting enzyme of sphingolipid biosynthesis is serine palmitoyltransferase (SPT), a multiprotein complex catalyzing the first step of sphingolipid *de novo* synthesis pathway (Hanada, 2003; Hornemann et al., 2007). Serine palmitoyltransferase long chain subunit 1 (SPTLC-1) is one subunit of SPT (Hanada, 2003; Hornemann et al., 2006). Inactivation of the *sptl-1* gene causes *C. elegans* unresponsive to mitochondrial dysfunction and inhibits antimycin-induced UPRmt induction (Liu et al., 2014). Hence, we speculate that the sphingolipids metabolism pathway also takes part in the UPRmt activation during the AD process.

In this study, we examined UPRmt related proteins levels in APP/PS1 mouse and SHSY5Y cells treated with Aβ to determine if the UPRmt contributes to AD pathogenesis. We exploited chemical drugs and small interfering RNAs to manipulate the mevalonate and sphingolipids biogenesis pathways, using drug or siRNA to evidence the involvement of these pathways in activating UPRmt.

## Materials and Methods

### Reagents and Preparation of Drugs

Amyloid β protein fragment 25–35 (Aβ_25–35_A4559) was purchased from Sigma-Aldrich. The Aβ_25–35_ was first dissolved in tri-distilled water to 1 mM and then incubated at 37°C for 7 days. The solution was aliquoted and stored at −20°C, until use. Simvastatin was purchased from MedChem Express (MCE, HY-17502).

### Antibodies

Antibodies specific for APP (126732), HtrA2/Omi (75982), β-Actin (11132), CLPP (124822), GAPDH (9485), respectively, were purchased from Abcam. Antibodies for LONP1 (15440), Hsp60 (15282), HMGCS-1 (17643), SPTLC-1 (15376) were obtained from Proteintech.

### Animals and Tissues

All procedures regarding the use of animals were conducted according to the guidelines and approved by the Ethical Committee for Animal Experiments of Shandong University. We used the APPsw/PS1dE9 double transgenic mice at the age of 3 and 9 months, age-matched C57BL/6 mice as a control. All mice were male (*n* = 10 per group) and purchased from Beijing HFK Bioscience Co., Limited (Beijing, China). When reaching the age of 3 and 9 months, mice were anesthetized by 10% of chloral hydrate and then sacrificed by cervical dislocation and decapitation. Fresh hippocampal tissues were harvested and stored at −80°C until use for studies. All applicable Shandong University and the ethical committee of the Second Hospital of Shandong University guidelines for the care and use of animals were followed.

### Cell Culture and Treatments

The human neuroblastoma cell line SHSY5Y was obtained from the Cell Resource Center, IBMS, CAMS/PUMC. Cells were cultured in RPMI-1640 medium (HyClone) supplemented with 10% fetal bovine serum (Gibco) at 37°C and 5% C0_2_. Cells at 50–70% confluence were treated with Aβ_25–35_, and or simvastatin. Control cells were cultured under normal conditions.

### Cell Viability Assay

Cells were seeded into 96-well plates. After culturing for 24 h, cells were treated with simvastatin or SPTLC-1 siRNA for 48 h and then treated with Aβ_25–35_ for another 4 h. After treatments, cells in each well were incubated with Cell Counting Kit 8 (CCK8) solution (MedCjemExpress) at 37°C for 3 h and then used for measuring the absorbance at 450 nm with a microplate reader (Thermo, Multiskan MK3, USA). Experiments were repeated for at least three times.

### SDS-PAGE and Western Blotting

The Hippocampal tissues and cells after treatments were homogenized in RIPA lysis buffer (Beyotime Biotechnology). Cell debris and nuclei were discarded after a centrifugation at 4°C 12,000 *g* for 10 min. The supernatants were collected and the protein concentration was measured with the bicinchoninic acid (BCA) method. After SDS-PAGE, proteins were blotted onto polyvinylidene difluoride (PVDF) membranes. Blots were blocked in 5% skimmed milk powder in TBST and incubated with corresponding primary antibodies at 4°C for 12–16 h. After extensive washes in TBST, blots were incubated with HRP conjugated secondary antibodies for 1–2 h at room temperature. Proteins were detected with ECL (enhanced chemiluminescence) regents.

### Transmission Electron Microscopy

Cells were collected and fixed in pre-cooled glutaraldehyde for at least 2 h. After postfixation in osmic acid solutions and sequential dehydration, cells were embedded in EPON812 resins. Ultrathin sections were cut and collected onto grids, stained with uranium and lead citrate, and observed under a JEM-1200EX electron microscope (JEOL, Tokyo, Japan).

### Measurement of ROS Levels

Intracellular reactive oxygen species (ROS) levels were detected using DCFH-DA (Beyotime, S0033), which is non-fluorescent and generates fluorescent signals after being oxidized into DCF in the presence of intracellular ROS. The day before experiments, cells were planted into 12-well plates and cultured for 24 h. After brief washes in PBS, cells were incubated with DCFH-DA (10 μmol/L) at 37°C for 20 min, then washed three times with RPMI-1640 medium. Fluorescence images were captured using a fluorescence microscope (OLYMPUS BX43) at excitation and emission wavelengths of 488 and 525 nm. The amount of ROS was quantified with the use of a fluorescence microplate reader. All experiments were repeated for at least three times.

### Transfection of siRNAs

SHSY5Y cells were transfected with target siRNAs using INTERFRERin (Polyplus-transfection Inc., New York, NY, USA). Corresponding scrambled siRNAs were used as controls. After transfection with siRNA for 48 h, cells were treated with or without Aβ as indicated and then collected cells for examinations.

### Statistical Analysis

All data were expressed as mean ± SEM. One-way analysis of variance (ANOVA) or Two-tailed student’s *t*-test was used for determining statistical significance. Data were analyzed using SPSS 20.0 (SPSS Inc., Chicago, IL, USA). A *p-*value of less than 0.05 was considered to be statistically significant.

## Results

### Aβ Activates UPRmt Responses

**Figure 1:**
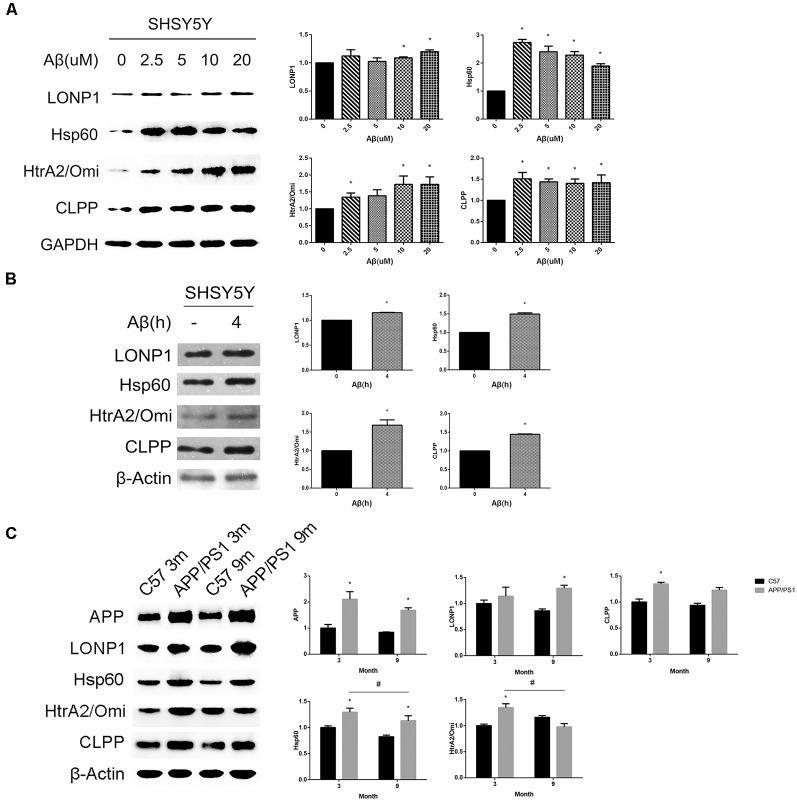
UPRmt response activated during the Alzheimer disease (AD) process *in vitro* and *in vivo*. **(A)** Western blot analysis of UPRmt related proteins level in SHSY5Y cells treated with different concentrations of Aβ_25–35_ for 24 h and quantification, GAPDH served as the internal control. **(B)** Western blot analysis of UPRmt related proteins level in SHSY5Y cells treated with 20 μM Aβ_25–35_ for 4 h, quantification and β-Actin served as the internal control. The data are mean ± SEM (*n* = 3, *p < 0.05 vs. control group). **(C)** Western blot analysis of UPRmt related proteins level in C57 and APP/PS1 transgenic mice at 3 and 9 months, quantification and β-Actin served as the internal control. The data are mean ± SEM (*n* = 10, *p < 0.05 vs. age-matched C57 mice group, ^#^*p* < 0.05).

To determine whether Aβ_25–35_ treatment can cause the above UPRmt reactions, we treated SHSY5Y cells with different concentrations of Aβ_25–35_ for 24 h and then examined expression levels of UPRmt related proteins. We found that expression levels of mitochondrial matrix chaperone Hsp60, AAA proteases CLPP and the IMS-localized quality control protease HtrA2/Omi were increased after exposure to 2.5, 10 and 20 μM Aβ_25–35_ for 24 h. Levels of Hsp60 and CLPP, but not HtrA2/Omi, were also significantly increased in cells treated with 5 μM Aβ_25–35_ for 24 h. Under the same conditions, the upregulation of the LONP1 protein was detected only in cells exposed to 10 and 20 μM Aβ_25–35_ (Figure 1A). We then investigated whether treatment with 20 μM Aβ_25–35_ for a shorter time period could sufficiently activate UPRmt. Our data showed that the expression levels of all the four proteins mentioned above were elevated in SHSY5Y cells after exposure to 20 μM Aβ_25–35_ for 4 h (Figure 1B).

To determine whether the UPRmt occurred in the brain of APPsw/PS1dE9 double transgenic mice, we first conducted biochemical studies to compare expression levels of UPRmt related proteins in hippocampi of 3 and 9 months old wildtype (WT) and APPsw/PS1dE9 transgenic mice, levels of APP in hippocampal lysates of both 3 and 9 months old transgenic mice was significantly increased compared with those in hippocampal lysates of age-matched WT mice (Figure 1C). Western blot analysis revealed that expression levels of Hsp60, CLPP and HtrA2/Omi were significantly increased in the hippocampus of 3 months old APPsw/PS1dE9 transgenic mice relative to those of 3 months old WT mice (Figure 1C). Under the same conditions, levels of the LONP1 protein were comparable in hippocampi of 3 months old WT and transgenic mice. However, levels of HtrA2/Omi and CLPP in hippocampal lysates of 9 months old transgenic mice were not different from those in hippocampal lysates of 9 months old WT mice, whereas levels of LONP1 and Hsp60 in the hippocampus of 9 months old transgenic mice were increased compared with those of age-matched control mice (Figure 1C). We also found that the expression levels of Hsp60 and HtrA2/Omi were significantly decreased in the hippocampus of 9 months old APPsw/PS1dE9 transgenic mice relative to those of 3 months old transgenic mice. Taken together, our *in vitro* and *in vivo* data suggest that UPRmt occurs in the process of AD pathological development.

### The Mevalonate Pathway is Involved in Aβ_25–35_ Evoked UPRmt in SHSY5Y Cells

**Figure 2:**
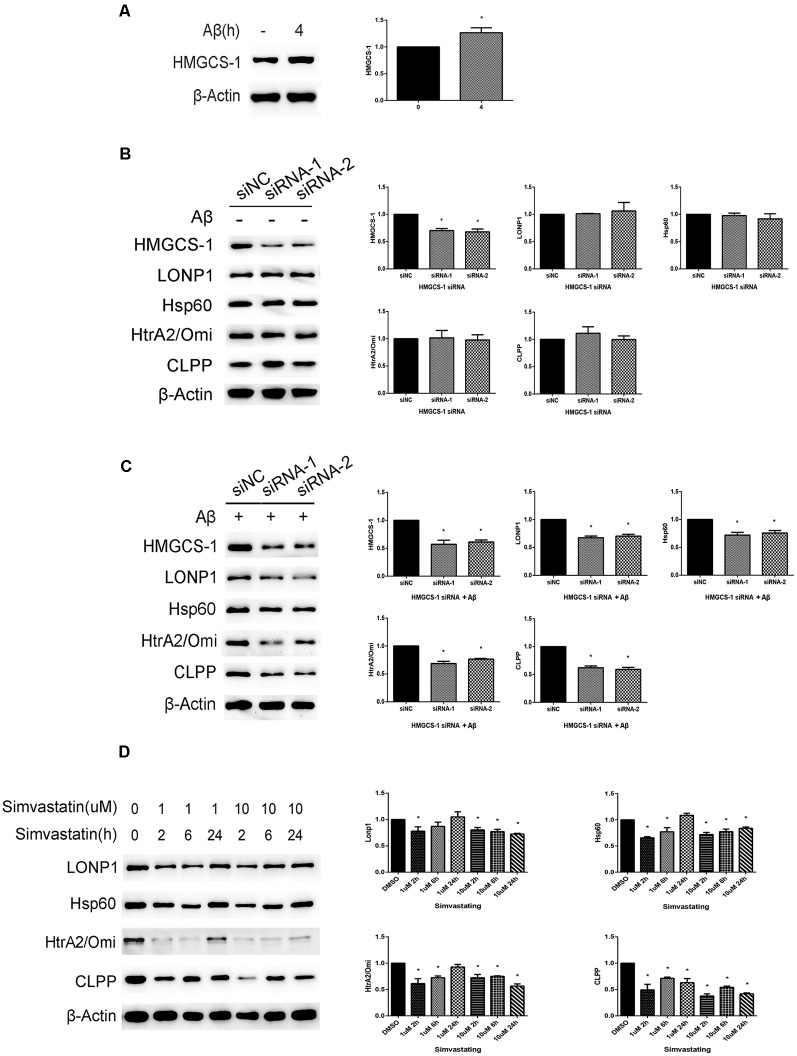
Inhibition of the mevalonate pathway by HMGCS-1 siRNAs or simvastatin reduces the UPRmt reaction induced by Aβ_25–35_ in SHSY5Y cells. **(A)** Western blot analysis of HMGCS-1 protein level in SHSY5Y cells treated with 20 μM Aβ_25–35_ for 4 h, quantification and β-Actin served as the internal control. The data are mean ± SEM (*n* = 3, *p < 0.05 vs. control group). **(B)** Western blot analysis of HMGCS-1 and UPRmt related proteins level in SHSY5Y cells transfected with scramble or HMGCS-1 siRNAs for 48 h, quantification and β-Actin served as the internal control. The data are mean ± SEM (*n* = 3, *p < 0.05 vs. siNC group). **(C)** Western blot analysis of HMGCS-1 and UPRmt related proteins level in SHSY5Y cells transfected with scramble or HMGCS-1 siRNAs for 48 h and 20 μM Aβ_25–35_ treatment for 4 h, quantification and β-Actin served as the internal control. The data are mean ± SEM (*n* = 3, *p < 0.05 vs. siNC group). **(D)** After treated with 1 μM and 10 μM simvastatin for different time courses, we detected the UPRmt related proteins level in SHSY5Y cell with 20 μM Aβ_25–35_ by western blot analysis, β-Actin served as the internal control.The data are mean ± SEM (*n* = 3, *p < 0.05 vs. DMSO group).

There was upregulated expression of HMGCS-1, an enzyme that mediates the first committed step of the mevalonate pathway, in cells treated with 20 μM Aβ_25–35_ for 4 h (Figure 2A). This observation led us to speculate that the mevalonate pathway might be involved in the activation of the UPRmt in cells treated with Aβ. To test this possibility, we treated SHSY5Y cells with HMGCS-1 siRNAs or with simvastatin to manipulate the mevalonate pathway. The expression level of HMGCS-1 was decreased in cells transfected with HMGCS-1 siRNAs for 48 h, compared with the HMGCS-1 level in cells transfected with scrambled siRNA. As shown in Figure 2B, in the absence of Aβ, there was no statistically significant difference in UPRmt related protein levels between cells transfected with scrambled siRNA and target siRNA. However, we found that after transfected with HMGCS-1 siRNAs, the expression levels of LONP1, Hsp60, HtrA2/Omi, and CLPP were significantly down-regulated in cells treated with 20 μM Aβ for 4 h (Figure 2C). We then detected whether simvastatin treatment could similarly dampen the effect of Aβ_25–35_ on the activation of the UPRmt reaction. We first treated cells with or without 1 μM or 10 μM of simvastatin for 2 h, 6 h, and 24 h, and then added 20 μM Aβ_25–35_ to cell culture media to treat cells for 4 h. The data in Figure 2D showed that except for the treatment with 1 μM simvastatin for 24 h, all other treatment conditions significantly decreased the expression of Hsp60 and HtrA2/Omi compared with the vehicle treatment. Compared with those in vehicle-treated cells, levels of CLPP in all of the simvastatin treatment conditions were decreased. Except for the group that 1 μM simvastatin treatment for 6 h and 24 h, expression of Lonp1 protein in other groups were decreased compared with control (Figure 2D). From the above, we can see that inhibition of the mevalonate pathway induced by HMGCS-1 siRNAs or simvastatin reduce the expression of UPRmt related proteins in SHSY5Y cells treated with Aβ_25–35_.

### Simvastatin Treatments Change the Cell Morphology and Mitochondrial Structure, Increase the Intracellular ROS Level and Aggravate the Cytotoxic Effect of Aβ_25–35_ in SHSY5Y Cells

**Figure 3:**
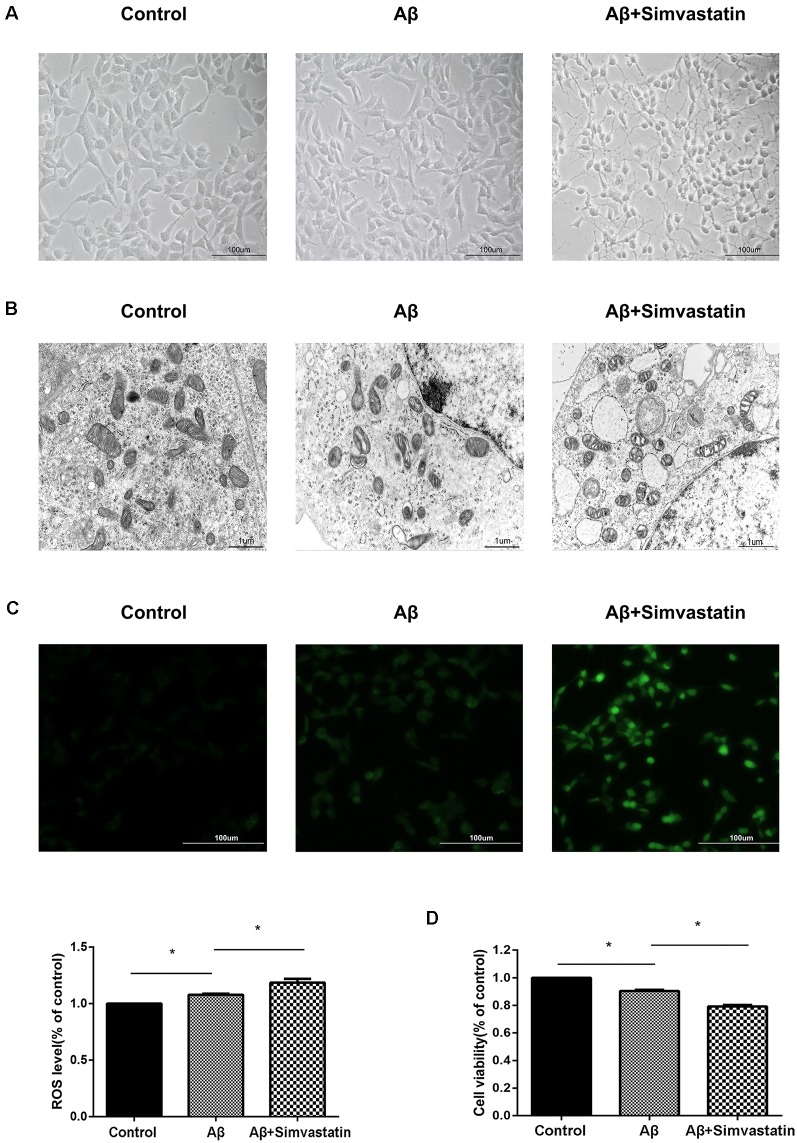
Simvastatin treatment changes the cell morphology and mitochondrial structure, increases the intracellular reactive oxygen species (ROS) level and aggravates the cytotoxic effect of Aβ_25–35_ in SHSY5Y cells. After treated with 10 μM simvastatin for 24 h and 20 μM Aβ_25–35_ for 4 h, the morphology of SHSY5Y cells were observed by a light microscope **(A)**, the mitochondrial structures were observed by an electron microscope **(B)**, the intracellular ROS level was measured by DCFH **(C)**, viability of the cells was examined by Cell Counting Kit 8 (CCK8) assay **(D)**. The data are mean ± SEM (*n* = 3, *p < 0.05).

During experiments, we observed a phenomenon with a microscope, that 24 h treatment of 10 μM simvastatin changed the cell’s morphology and promoted the apoptosis of cells treated with Aβ_25–35_ (Figure 3A). The electron micrograph also showed that the simvastatin treatment aggravates the morphological changes of mitochondrial, such as mitochondrial vacuolation, disorganization, and reduction of the crista (Figure 3B). We further tested the intracellular ROS level by use of the ROS-sensitive fluorescent probe DCFH-DA, the fluorescence images and the amount of ROS showed that simvastatin treatment aggravated the increased intracellular ROS level induced by Aβ_25–35_ (Figure 3C). The cells viability was tested with the CCK8 method. As shown in Figure 3D, pretreatment with 10 μM simvastatin for 24 h aggravated the decrease of cell viability induced by Aβ_25–35_.

### The Sphingolipid Biosynthesis Pathway is Involved in Aβ_25–35_ Evoked UPRmt in SHSY5Y Cells

**Figure 4:**
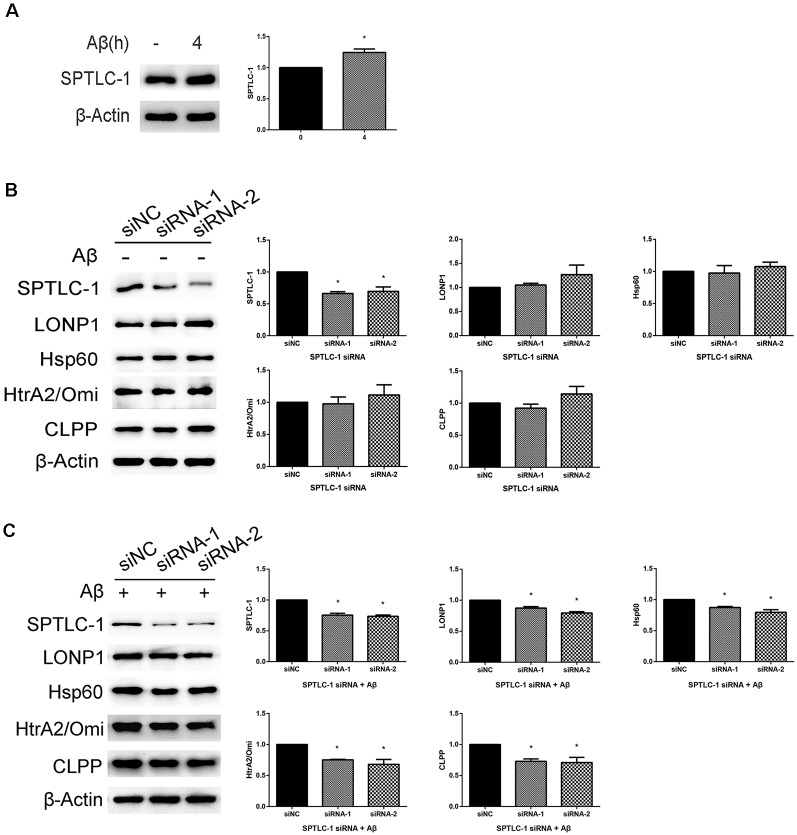
Inhibition of the sphingolipid biosynthesis pathway by serine palmitoyltransferase long chain subunit 1 (SPTLC-1) siRNAs reduces the UPRmt reaction induced by Aβ_25–35_ in SHSY5Y cells. **(A)** Western blot analysis of SPTLC-1 protein level in SHSY5Y cells treated with 20 μM Aβ_25–35_ for 4 h, quantification and β-Actin served as the internal control. The data are mean ± SEM (*n* = 3, *p < 0.05 vs. control group). **(B)** Western blot analysis of SPTLC-1 and UPRmt related proteins level in SHSY5Y cells transfected with scramble or SPTLC-1 siRNAs for 48 h, quantification and β-Actin served as the internal control. The data are mean ± SEM (*n* = 3, *p < 0.05 vs. siNC group). **(C)** Western blot analysis of SPTLC-1 and UPRmt related proteins level in SHSY5Y cells transfected with scramble or SPTLC-1 siRNAs for 48 h and 20 μM Aβ_25–35_ treatment for 4 h, quantification and β-Actin served as the internal control. The data are mean ± SEM (*n* = 3, *p < 0.05 vs. siNC group).

In a previous study, RNAi mediated inactivation of the sptl-1 gene, which encodes SPT, disrupted response to mitochondrial dysfunction in *C. elegans*. To detect whether it happens in SHSY5Y cells treated with Aβ_25–35_, we used the SPTLC-1 siRNAs to hinder sphingolipid biosynthesis. As shown in Figure 4A, treatment of cells with 20 μM Aβ_25–35_ for 4 h led to the upregulation of SPTLC-1. The expression level of SPTLC-1 was decreased in cells transfected with SPTLC-1 siRNAs for 48 h, compared with the SPTLC-1 level in cells transfected with scrambled siRNA. As shown in Figure 4B, in the absence of Aβ, there was no statistically significant difference in UPRmt related protein levels between cells transfected with scrambled siRNA and target siRNA. However, we found that after transfected with SPTLC-1 siRNAs, the protein levels of LONP1, Hsp60, HtrA2/Omi and CLPP were both decreased in cells treated with 20 μM Aβ for 4 h (Figure 4C). Thus, the Inhibition of the sphingolipid biosynthesis pathway by SPTLC-1 siRNAs decreases the UPRmt related proteins level in SHSY5Y cells treated with Aβ_25–35_.

### Inhibition of the Sphingolipid Biosynthesis Pathway by SPTLC-1 siRNAs Changes the Mitochondrial Structure, Increases the Intracellular ROS Level and Aggravates the Cytotoxic Effect of Aβ_25–35_ in SHSY5Y Cells

**Figure 5:**
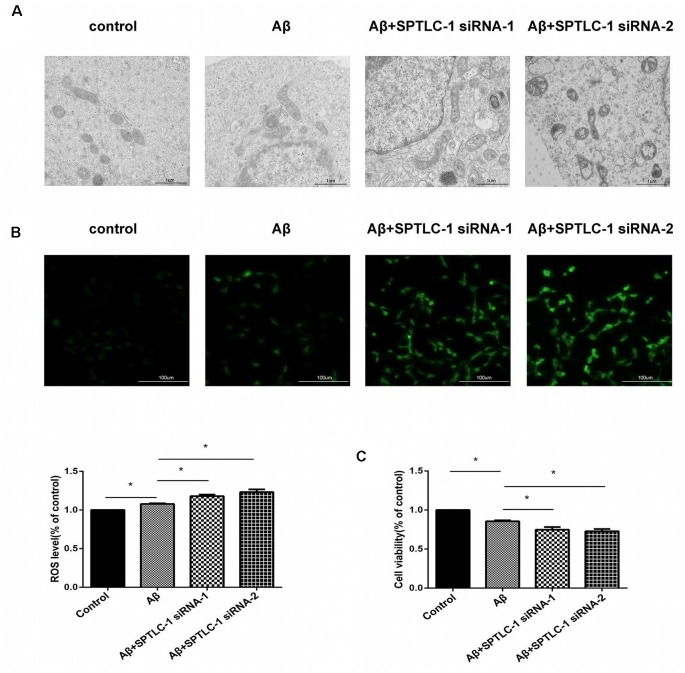
Inhibition of the sphingolipid biosynthesis pathway by SPTLC-1 siRNAs changes the mitochondrial structure, increases the intracellular ROS level and aggravates the cytotoxic effect of Aβ_25–35_ in SHSY5Y cells. After transfected with SPTLC-1 siRNAs for 48 h and 20 μM Aβ_25–35_ for 1 h, the mitochondrial structure of SHSY5Y cells was observed by a electron microscope **(A)**, the intracellular ROS level was measured by DCFH **(B)**, viability of the cells was examined by CCK8 assay **(C)**. The data are mean ± SEM (*n* = 3, *p < 0.05).

To determine whether inhibition of the sphingolipid biosynthesis pathway can cause a similar effect like simvastatin, we tested the cells’ mitochondrial structure, intracellular ROS level, and cell viability after transfected with SPTLC-1 siRNAs. As shown in Figure 5A, the Inhibition of the sphingolipid biosynthesis pathway by SPTLC-1 siRNAs aggravated the abnormalities in mitochondrial morphology, such as mitochondrial vacuolation and reduction of the crista. The fluorescence images and the amount of ROS also showed that Aβ_25–35_ increased the intracellular ROS level and SPTLC-1 siRNAs transfection aggravated the ROS accumulation (Figure 5B). The cells viability tested by the CCK8 method also showed that SPTLC-1 siRNAs transfection aggravated the decrease of cell viability induced by Aβ_25–35_ (Figure 5C).

## Discussion

This study showed that the UPRmt was activated in SHSY5Y cells with Aβ_25–35_ treatment and APPsw/PS1dE9 transgenic mice, and inhibition of the mevalonate and sphingolipid biosynthesis pathway disrupted the activation of UPRmt in SHSY5Y cells induced by Aβ_25–35_. We further found that inhibition of these two pathways through simvastatin and SPTLC-1 siRNAs aggravated the mitochondrial injury, ROS accumulation and the decrease of cell viability in SHSY5Y cells induced by Aβ_25–35_.

Numerous diseases are associated with UPRmt, including spastic paraplegia, Parkinson’s disease, Friedreich’s ataxia and cancer (Haynes and Ron, 2010). Most reports about the unfolded protein response in AD were focused on the endoplasmic reticulum, but studies about the UPRmt were little except for the research mentioned above (Beck et al., 2016). So we tested the UPRmt reaction in cell and mice models of AD in this research. In cell models, all of the four UPRmt related proteins were upregulated in SHSY5Y cells treated with 10 μM or 20 μM Aβ_25–35_ for 24 h. As shown in results, the UPRmt related proteins Hsp60, HtrA2/Omi and CLPP were upregulated in APPsw/PS1dE9 transgenic mice at 3 months, while to 9 months, Hsp60 and LONP1 proteins were increased compared with age-matched WT mice. Thus, we conclude that the UPRmt response was activated during the AD process *in vitro* and *in vivo*.

Among the UPRmt related proteins, LONP1 as a mitochondrial matrix protease, maintain the mitochondrial homeostasis by degrading misfolded or oxidized polypeptides. As shown in Figure 1A, the LONP1 protein level was unchanged after treated the SHSY5Y cell with 2.5 or 5 μM Aβ_25–35_ for 24 h, but increased after 10 or 20 μM Aβ_25–35_ treatment. Combined with the results in the mouse model, the LONP1 protein level was unchanged in APPsw/PS1dE9 transgenic mice at 3 months but increased at 9 months compared with age-matched WT mice. Hence we speculate that the activation of LONP1 protein may need a prolonged or a large dose of Aβ stimulation, but further research is needed to confirm this. From the result in Figure 1C, we found that the expression levels of Hsp60 and HtrA2/Omi were significantly decreased in the hippocampus of 9 months old APPsw/PS1dE9 transgenic mice relative to those of 3 months old transgenic mice. Compared with age-matched WT mice, the UPRmt related proteins HtrA2/Omi and CLPP were upregulated in APPsw/PS1dE9 transgenic mice at 3 month but unchanged at 9 month, which suggest that the UPRmt activation decreased with age during theAD process. This is consistent with *Sorrentino’s research* that several UPRmt genes were down-regulated in the cortex samples of 9 months old AD mice compared with those of 6 months old AD mice (Sorrentino et al., 2017). Another research about the endoplasmic reticulum UPR response in Down syndrome also found that the UPR was selectively activated at 3 months, but restored to basal controls at 9 months (Lanzillotta et al., 2018). So we conclude that the UPRmt response is activated during the AD process in mice and cell models, but levels are altered in AD mice at different time points.

The mevalonate pathway is a key metabolic cascade that catalyzes the synthesis of sterol and nonsterol isoprenoids, which plays important effects on many cellular processes and physiological states (Goldstein and Brown, 1990). The HMG-CoA synthase HMGCS-1 and the HMG-CoA reductase HMGCR mediate this pathway as mentioned above, whereas stains as cholesterol-lowering drugs act by inhibiting the HMGCR. In our study, the HMGCS-1 protein level was increased in SHSY5Y cells after treated with 20 μM Aβ_25–35_ for 4 h, and after inhibiting the mevalonate pathway by HMGCS-1 siRNA or simvastatin in SHSY5Y cells treated with Aβ_25–35_, the expression of UPRmt related proteins were decreased. This is consistent with the researches in *C. elegans* that the UPRmt upregulates the expression of HMGS-1 (Oks et al., 2018), and inhibition of the mevalonate pathway through the *hmgr-1* deletion mutant, *hmgs-1* gene inactivation or statins prevents the activation of UPRmt (Liu et al., 2014; Ranji et al., 2014). We also found that inactivation of HMGCS-1 by siRNAs has no effect on the UPRmt reaction in SHSY5Y cells without Aβ treatment. From the above, our research provides evidence that the mevalonate pathway is necessary to the activation of UPRmt induced by Aβ and this effect of the mevalonate pathway was Aβ-dependent.

Several reports have revealed a novel connection between the sphingolipid biosynthesis and the endoplasmic reticulum UPR activation (Spassieva et al., 2009; Lépine et al., 2011; Epstein et al., 2012), but none was about the correlations between induction of UPRmt and the ceramide synthesis pathway in AD. As shown in our results, the SPTLC-1 protein level is increased in SHSY5Y cells after treated with 20 μM Aβ_25–35_ for 4 h. Inhibition of the ceramide synthesis by SPTLC-1 siRNA decreased the UPRmt related proteins levels in SHSY5Y cells treated with Aβ_25–35_ transiently, which provides evidence for an involvement of ceramide synthase SPTLC-1 in the activation of UPRmt response in AD cell models. The result is consistent with the researches in *C. elegans* that the UPRmt upregulates the transcript level of *sptl-1*, and inhibition of the sphingolipid biosynthesis pathway through the *sptl-1* gene inactivation prevents the activation of UPRmt (Liu et al., 2014). Ceramide, which is the core structure of all complex sphingolipids, serves as the second messenger that regulates diverse cellular processes such as growth, differentiation, and apoptosis (Pettus et al., 2002). Ceramide levels can be reduced by inhibition of SPT both *in vitro* and *in vivo* (Hojjati et al., 2005; Holland et al., 2007). It have been proved that ceramide levels were increased at the earliest stage during the AD process (Han et al., 2002; Katsel et al., 2007; Mielke et al., 2010). Studies in patients with sporadic AD also showed that the levels of ceramide and SPT protein including SPTLC-1 were significantly elevated compared with control (Geekiyanage and Chan, 2011). SPT activity has proved increasing in dealing with various stimuli in cell research (Perry et al., 2000; Scarlatti et al., 2003). We also found that inactivation of SPTLC-1 by siRNAs has no effect on the UPRmt reaction in SHSY5Y cells without Aβ treatment. Thus, we conclude that Aβ_25–35_ treatment increases the protein levels of SPTLC-1, and the sphingolipid biosynthesis pathway is necessary to the activation of UPRmt induced by Aβ and this effect of the mevalonate pathway was Aβ-dependent.

As shown in our results, inhibition of the mevalonate pathway or the sphingolipid biosynthesis pathway can prevent the activation of UPRmt, change the mitochondrial structure, increase the intracellular ROS level and aggravate the cytotoxic effect of Aβ_25–35_ in SHSY5Y cells, which suggest that the UPRmt can protect SHSY5Y cells from the damage of Aβ_25–35_. The UPRmt has been proved cytoprotective effects in several reports (Baker et al., 2012; Runkel et al., 2013; Lamech and Haynes, 2015), and activation of the UPRmt response can also extend lifespan (Durieux et al., 2011; Houtkooper et al., 2013). Therefore, our research provides new evidence for the UPRmt cytoprotective effect during the AD process. It also provides a new sight for us whether enhancing or artificial activation of UPRmt can protect cells from the cytotoxic effect of Aβ and works on the treatment of AD. But several reports have revealed that the prolonged or over activation of the UPRmt has an adverse effect like shortening the lifespan of *C. elegans* (Bennett et al., 2014), perturb mitochondrial function (Lamech and Haynes, 2015; Lin et al., 2016). So further research is needed on whether enhanced UPRmt activation can be used in the treatment of AD because the intensity and duration of the response should be considered.

Together, these observations imply that the UPRmt activated in AD cell and mice models, but the levels vary according to the ages of mice. We further observed that the mevalonate and sphingolipid biosynthesis pathways were necessary for the activation of UPRmt in AD. When inhibiting the two pathways, the cytotoxic effects of Aβ_25–35_ in SHSY5Y cells were aggravated. Hence our research helps us understanding the role of UPRmt in the pathogenesis of AD, which may provide new sights for the treatment of AD.

## Data Availability Statement

All datasets generated for this study are included in the article.

## Ethics Statement

The animal study was reviewed and approved by the Ethical Committee for Animal Experiments of the Second Hospital of Shandong University.

## Author Contributions

JB, PW, and YS designed the research. YS performed the experiments. MD, ZX, and XL provided technical support and helped with mouse dissections. HY, SJ, SX, ZZ, YW, DW, LX, and XZ analyzed the data. JB, PW, and YS wrote the manuscript. All authors read and approved the final manuscript.

## Conflict of Interest

The authors declare that the research was conducted in the absence of any commercial or financial relationships that could be construed as a potential conflict of interest.

## Abbreviations

AD: Alzheimer disease
Aβ: Amyloid β-peptide
APP: Amyloid Precursor Protein
UPRmt: Mitochondrial unfolded protein response
HtrA2/Omi: High-temperature requirement protein A2/Omi
Hsp60: Heat shock protein 60
CLPP: Caseinolytic protease P
LONP1: Lon peptidase 1
HMG-CoA: 3-hydroxy-3-methyl-glutaryl-CoA
HMGCS-1: Human hydroxymethylglutaryl-CoA synthase 1
SPT: serine palmitoyltransferase
SPTLC-1: Serine palmitoyltransferase long chain subunit 1
CCK8: Cell Counting Kit 8
BCA: Bicinchoninic acid
PVDF: Polyvinylidene difluoride
ROS: Reactive oxygen species
WT: Wildtype
IMS: Intermembrane space.
